# Supplementary material for: Self-Reported Measures of Periodontitis in a Portuguese Population: A Validation Study
Source: J Pers Med. 2022 Aug 14;12(8):1315. doi: 10.3390/jpm12081315 (PMC9410440; doi:10.3390/jpm12081315)
Supplement: Supplementary file 1 [file jpm-12-01315-s001.zip › jpm-1806079-supplementary.pdf]

Journal of Personalized Medicine

Supplementary information

Self-Reported Measures of Periodontitis in a Portuguese Population: A Validation Study

Vanessa Machado* [1,2,†], **Patrícia Lyra** [1,†], **Catarina Santos** [1], **Luís Proença** [1,2,3], **José João Mendes** [1,2], **João Botelho** [1,2]

[1] Clinical Research Unit, Centro de Investigação Interdisciplinar Egas Moniz (CiiEM), Egas Moniz—Cooperativa de Ensino Superior, 2829-511 Almada, Portugal
[2] Evidence-Based Hub, CiiEM, Egas Moniz—Cooperativa de Ensino Superior, 2829-511 Almada, Portugal;
[3] Quantitative Methods for Health Research, CiiEM, Egas Moniz—Cooperativa de Ensino Superior, 2829-511 Almada, Portugal.
† These authors contributed equally to this work as first authors.
* Correspondence: vmachado@egasmoniz.edu.pt (V.M.).

Supplementary Table S1. Responses to questions stratified by periodontitis status according to the EFP/AAP 2018 and CDC/AAP 2012 case definitions and respective severe staging.

Variable	Total (N=103)	EFP/AAP 2018 Periodontitis (n=63)	EFP/AAP 2018 Severe Periodontitis (n=39)	CDC/AAP 2012 Periodontitis (n=71)	CDC/AAP 2012 Severe Periodontitis (n=56)
**Gum disease**					
Yes	56 (54.4)	35 (55.6)	39 (100)	32 (45.1)	30 (53.6)
No	39 (37.9)	22 (34.9)	0 (0.0)	39 (54.9)	26 (46.4)
Refused ∣ Don't Know	8 (7.8)	6 (9.5)	0 (0.0)	0 (0.0)	0 (0.0)
**Teeth/gum health**					
Excellent ∣ Very good	19 (18.4)	0 (0.0)	0 (0.0)	1 (1.4)	0 (0.0)
Good ∣ Fair ∣ Poor	84 (81.6)	63 (100)	39 (100)	70 (98.6)	56 (100)
Refused ∣ Don't Know	0 (0.0)	0 (0.0)	0 (0.0)	0 (0.0)	0 (0.0)
**Had gum treatment**					
Yes	84 (81.6)	63 (100)	39 (100)	70 (98.6)	56 (100)
No	19 (18.4)	0 (0.0)	0 (0.0)	1 (1.4)	0 (0.0)
Refused ∣ Don't Know	0 (0.0)	0 (0.0)	0 (0.0)	0 (0.0)	0 (0.0)
**Loose tooth**					
Yes	36 (35)	33 (52.4)	24 (61.5)	33 (46.5)	30 (53.6)
No	67 (65)	30 (47.6)	15 (38.5)	38 (53.5)	26 (46.4)
Refused ∣ Don't Know	0 (0.0)	0 (0.0)	0 (0.0)	0 (0.0)	0 (0.0)
**Lost bone**					
Yes	40 (38.8)	37 (58.7)	26 (66.7)	40 (56.3)	36 (64.3)
No	62 (60.2)	26 (41.3)	13 (33.3)	31 (43.7)	20 (35.7)
Refused ∣ Don't Know	1 (1.0)	0 (0.0)	0 (0.0)	0 (0.0)	0 (0.0)
**Tooth does not look right**					
Yes	55 (53.4)	44 (69.8)	30 (76.9)	47 (66.2)	40 (71.4)
No	48 (46.6)	19 (30.2)	9 (23.1)	24 (33.8)	16 (28.6)
Refused ∣ Don't Know	0 (0.0)	0 (0.0)	0 (0.0)	0 (0.0)	0 (0.0)
**Floss use**					
Never	47 (45.6)	31 (49.2)	20 (51.3)	34 (47.9)	27 (48.2)
1-7 times	56 (54.4)	32 (50.8)	19 (48.7)	37 (52.1)	29 (51.8)
Refused ∣ Don't Know	0 (0.0)	0 (0.0)	0 (0.0)	0 (0.0)	0 (0.0)
**Gum bleeding**					
Yes	15 (14.6)	15 (23.8)	9 (23.1)	15 (21.1)	12 (21.4)
No	88 (85.4)	48 (76.2)	30 (76.9)	56 (78.9)	44 (78.6)
Refused ∣ Don't Know	0 (0.0)	0 (0.0)	0 (0.0)	0 (0.0)	0 (0.0)
**Gum bleeding last 3 months**					
Very often ∣ Fairly often ∣ Sometimes	44 (42.7)	31 (49.2)	19 (48.7)	33 (46.5)	25 (44.6)
Never ∣ Hardly ever	59 (57.3)	32 (50.8)	20 (51.3)	38 (53.5)	31 (55.4)
**Loose teeth loss**					
Yes	22 (21.4)	21 (33.3)	14 (35.9)	21 (29.6)	20 (35.7)
No	81 (78.6)	42 (66.7)	25 (64.1)	50 (70.4)	36 (64.3)
Refused ∣ Don't Know	0 (0.0)	0 (0.0)	0 (0.0)	0 (0.0)	0 (0.0)
**Gum pain**					
Yes	24 (23.3)	18 (28.6)	10 (25.6)	19 (26.8)	14 (25)
No	79 (76.7)	45 (71.4)	29 (74.4)	52 (73.2)	42 (75)

Refused ∣ Don't Know	0 (0.0)	0 (0.0)	0 (0.0)	0 (0.0)	0 (0.0)
**Gum retraction**					
Yes	34 (33.0)	27 (42.9)	21 (53.8)	32 (45.1)	25 (44.6)
No	67 (65.0)	35 (55.6)	17 (43.6)	38 (53.5)	30 (53.6)
Refused ∣ Don't Know	2 (1.9)	1 (1.6)	1 (2.6)	1 (1.4)	1 (1.8)
**Roots visible**					
Yes	31 (30.1)	26 (41.3)	19 (48.7)	29 (40.8)	23 (41.1)
No	72 (69.9)	37 (58.7)	20 (51.3)	42 (59.2)	33 (58.9)
Refused ∣ Don't Know	0 (0.0)	0 (0.0)	0 (0.0)	0 (0.0)	0 (0.0)
